# Monitoring bone and soft-tissue tumors after carbon-ion radiotherapy using ^18^F-FDG positron emission tomography: a retrospective cohort study

**DOI:** 10.1186/s13014-015-0571-9

**Published:** 2015-12-21

**Authors:** Takashi Yanagawa, Kenichi Saito, Hiroki Kiyohara, Tatsuya Ohno, Takashi Nakano, Kenji Takagishi

**Affiliations:** Department of Orthopaedic Surgery, Gunma University Graduate School of Medicine, 3-39-22, Showa, Maebashi, Gunma 371-8511 Japan; Gunma University Heavy Ion Medical Center, Gunma University, 3-39-22, Showa, Maebashi, Gunma 371-8511 Japan

**Keywords:** FDG-PET, Carbon ion radiotherapy, Sarcoma, Bone tumor, Soft-tissue tumor, Heavy ion radiotherapy

## Abstract

**Background:**

The results of treatment for malignant bone and soft-tissue tumors arising from the deep trunk and pelvis are still not acceptable due to the relatively high recurrence and low overall survival rates. Recently, carbon ion radiotherapy (CIRT) was applied for several malignancies, including bone and soft-tissue sarcomas, and provided favorable results. However, it has been unclear what modalities should be used for evaluating the response and for the follow-up of these patients. Here, we analyzed the methods used to predict local recurrence and to find local failures or metastases.

**Methods:**

We analyzed 37 patients with bone and soft-tissue tumors who received CIRT at our institute. The patients were examined with FDG positron emission tomography (PET) and enhanced MRI before and three months after CIRT. The pre-treatment maximum standardized uptake value (SUVmax), and that three months after treatment, the difference between the pre- and post-CIRT SUVmax, the ratio of the post- to pre-SUVmax in FDG-PET and the size of the tumors were evaluated as predictors for local recurrence. FDG-PET and enhanced MRI were used to detect local recurrence.

**Results:**

Local recurrence appeared in 10 cases after CIRT. Nine of the 10 lesions (90.0 %) were detected with FDG-PET, while enhanced MRI detected just 50.0 % of the recurrences. One case of local recurrence, in which the lesion was negative on FDG-PET, was detected using enhanced MRI. A receiver operating characteristic curve analysis showed that neither the SUVmax on FDG-PET nor the tumor size before or three months after CIRT could be used to predict local recurrence.

**Conclusions:**

The combination of FDG-PET and enhanced MRI is recommended to detect local recurrence for patients with sarcomas who have received CIRT; however, no parameters obtained during the examinations performed before and three months after CIRT accurately predicted the development of local recurrence.

## Background

Treatment of malignant bone and soft-tissue tumors arising from the deep trunk and pelvis is usually difficult because of their anatomical characteristics and relatively huge size due to a delayed diagnosis. Tumors located in the trunk or pelvis are known to carry a risk for local recurrence after surgical resection in patients with malignant soft-tissue tumors [1, 2], and the overall survival rate and local control rate of osteosarcoma in the trunk and pelvis were inferior to those of the extremities [3–5].

Our institute started performing carbon ion radiotherapy (CIRT) in 2010, and has treated various kinds of tumors, including sarcomas originating from the trunk and pelvis, for which surgical treatment is difficult or impossible [6]. The carbon ion beam exhibits a higher linear energy transfer to the target lesions than does a photon beam, resulting in the induction of effective damage to tumor cells [7]. Recently, CIRT was reported to achieve favorable results for the treatment of malignant bone and soft-tissue sarcomas located around the trunk and pelvis, especially for chordoma [8–10].

However, there remain issues associated with the use of CIRT, one of which is how to evaluate the tumor response just after treatment. Due to the lacking of surgical specimens, a pathological evaluation is impossible in the inoperable cases treated with CIRT. Performing a needle biopsy after CIRT may partially resolve this problem; however, the small amount of samples collected during such biopsies is associated with a risk of overlooking recurrence. Therefore, modalities that can precisely evaluate the tumor response after CIRT are required.

One candidate modality is 2-deoxy-2-[F-18] fluoro-D-glucose positron emission tomography (FDG-PET), which has been used to evaluate the response of sarcomas to neoadjuvant chemotherapy. The FDG accumulation, presented as the standardized uptake value (SUV), after treatment or the ratio of the post- to pre-treatment SUV were reported to correlate with the proportion of tumor necrosis [11]. Evilevitch et al. showed that a reduction of FDG uptake more accurately predicted the histopathological response of high-grade soft-tissue sarcomas to neoadjuvant chemotherapy than did a change in tumor size [12].

The purpose of this study was to analyze the modalities used to evaluate the effects of CIRT on bone and soft-tissue sarcomas and identify the best method for detecting local recurrence and distant metastasis after CIRT.

## Methods

### Patients

The patients who had bone and soft-tissue tumors in the trunk, and whose tumors were unresectable or difficult to surgically treat without causing severe dysfunction were eligible for CIRT. We reviewed 55 patients with bone and soft-tissue tumors who were treated with CIRT at our institute from November 2010 to January 2014, and who were followed-up for at least one year. Among them, 13 patients were excluded because they did not receive pre- or post-treatment FDG-PET/CT, three patients were excluded because chemotherapy was started within 1 month after CIRT, and this factor might affect the SUV of FDG-PET, and two patients who were treated with postoperative CIRT were excluded because their tumors were not radiologically detected prior to treatment. The remaining 37 patients were analyzed in this study. The patients were examined with FDG-PET/CT and enhanced MRI before and three months after CIRT. After that, examinations were performed at three to six month intervals to screen for metastases and recurrences. Screening for distant metastases was performed using FDG-PET/CT because of its usefulness in detecting musculoskeletal metastases [13]. The regions treated with CIRT were the sacrum in 10 patients, intrapelvic region in six, retroperitoneum in four, acetabulum in three, cervical spine in three, and other locations in the other patients.

Institutional Review Board approval was obtained on April 28, 2010 with registration number 765. Written informed consent was obtained from all subjects (patients) in this study.

### CIRT

The details of the CIRT technique and the modalities used at the Gunma University Heavy Ion Medical Center (GHMC) have been reported previously [6]. Briefly, the carbon ion beams generated by the GHMC had maximum accelerated energies of 400 million electron volts per nucleon (MeV/n). The planning target volume included the clinical target volume plus a 5 mm safety margin. The dose was expressed as the Gy (RBE) (the carbon physical dose [Gy] x relative biologic effectiveness [RBE]). The treatment was performed once a day at doses ranging from 64.0 to 70.4 Gy (RBE) for a total of 16 fixed fractions. The patients who received pre-CIRT chemotherapy received CIRT at least four weeks after the last dose of chemotherapy.

### FDG-PET/CT

The patients fasted for 6 h before the intravenous injection of ^18^F-FDG (5 MBq/kg). FDG PET/CT images were acquired 1 h after injection with the Discovery STE PET/CT scanner (GE Healthcare, Milwaukee WI) and Biograph 16 PET/CT scanner (Siemens, Malvem, PA). All PET images were independently interpreted by two experienced doctors of nuclear medicine. Functional images of the SUV were produced for a semiquantitative analysis. The SUV was defined as follows: SUV = radioactive concentration in the region of interest (MBq/g)/injected dose (MBq)/patient’s body weight (g). The SUVmax was defined as the peak SUV within the region of interest.

### Evaluation of predictors of interest

Several values have previously been utilized for predicting the effects of chemotherapy on bone and soft-tissue sarcomas. According to previous reports, the pre-treatment SUVmax (pre-Tx SUVmax), that three months after treatment (post-Tx SUVmax), the difference between the pre- and post-Tx SUVmax (ΔSUVmax) and the ratio of the post-Tx SUVmax to pre-Tx SUVmax (ratio of SUVmax) in FDG-PET/CT were evaluated as predictors. In addition, the maximum dimension of the pre-and post-treatment tumors were also evaluated. The pre-treatment tumor size (pre-Tx tumor size), post-treatment tumor size (post-Tx tumor size), difference in tumor size between the pre- and post-Tx measurements (Δtumor size) and the ratio of the post-Tx tumor size to the pre-Tx size (ratio of tumor size) were defined as parameters. The tumor size was measured with MRI, and the response to the treatment was classified into four groups: complete response, partial response, progressive disease and stable disease according to the RECIST version 1.1 [14]. We regarded local recurrence as an indicator of the effects of CIRT in the current study, because unlike an evaluation of neoadjuvant chemotherapy, a pathological analysis could not be used to evaluate the effects of the CIRT on the tumors. The findings regarding local recurrence were an increase or emergence of FDG accumulation in FDG-PET/CT and a gadolinium-enhanced lesion on MRI without indications for inflammatory diseases, such as an infection.

### Statistical analysis

The sensitivity and specificity for the prediction of local recurrence by each predictor of interest were calculated, and receiver operating characteristic (ROC) curves were created. Using the ROC curves, the propensity of the predictors to predict local recurrence was compared to that of the reference, which had an area under the curve (AUC) of 0.5. The cut-off point that minimized the distance between the point (0, 1) on the upper left hand corner of the ROC space and ROC curve was defined as optimal. The sample size was based on a significance level of 5 %, a power level of 80 %, an expected AUC = 0.8 and a 25 % recurrence rate after CIRT, which was estimated from previous reports [8, 15]. The required sample size was 36. The differences in the values of the predictors between the recurrence and no recurrence groups were analyzed with t-tests. The difference between the pre-and post-Tx SUVmax by FDG-PET/CT and the pre- and post-Tx tumor sizes were analyzed with paired t-tests. The statistical analyses for sample size estimation were performed using the MedCalc Statistical Software program, version 13.3.3 (MedCalc Software bvba, Ostend, Belgium; http://www.medcalc.org; 2014), and the others were performed with the SPSS software program (version 22.0; IBM Corporation, Somers, NY).

## Results

### Characteristics of the patients

The patients comprised 25 males and 12 females, and the mean age at CIRT was 62.9 years (range: 34 to 84 years). The mean follow-up period after CIRT was 25.9 months (range: 12 to 49 months). The pathological diagnoses are presented in Table 1. One patient with dedifferentiated liposarcoma underwent palliative chemotherapy more than one month after CIRT. Three patients died of the disease and one died of another disease. All patients could receive FDG-PET/CT before CIRT and three months after CIRT, while two patients could not be examined with enhanced MRI because one had wires in the sternum near the lesions and one missed the examination three months after CIRT.

**Table 1:** Pathological diagnosis

chordoma	11
undifferentiated sarcoma	9
chondrosarcoma	4
dedifferentiated liposarcoma	3
desmoid type fibromatosis	2
myxofibrosarcoma	2
malignant peripheral nerve sheath tumor	2
rhabdomyosarcoma	1
malignant solitary fibrous tumor	1
sclerosing epithelioid fibrosarcoma	1
well-differentiated liposarcoma	1
total	37

### Local recurrence and metastasis

**Fig. 1:**
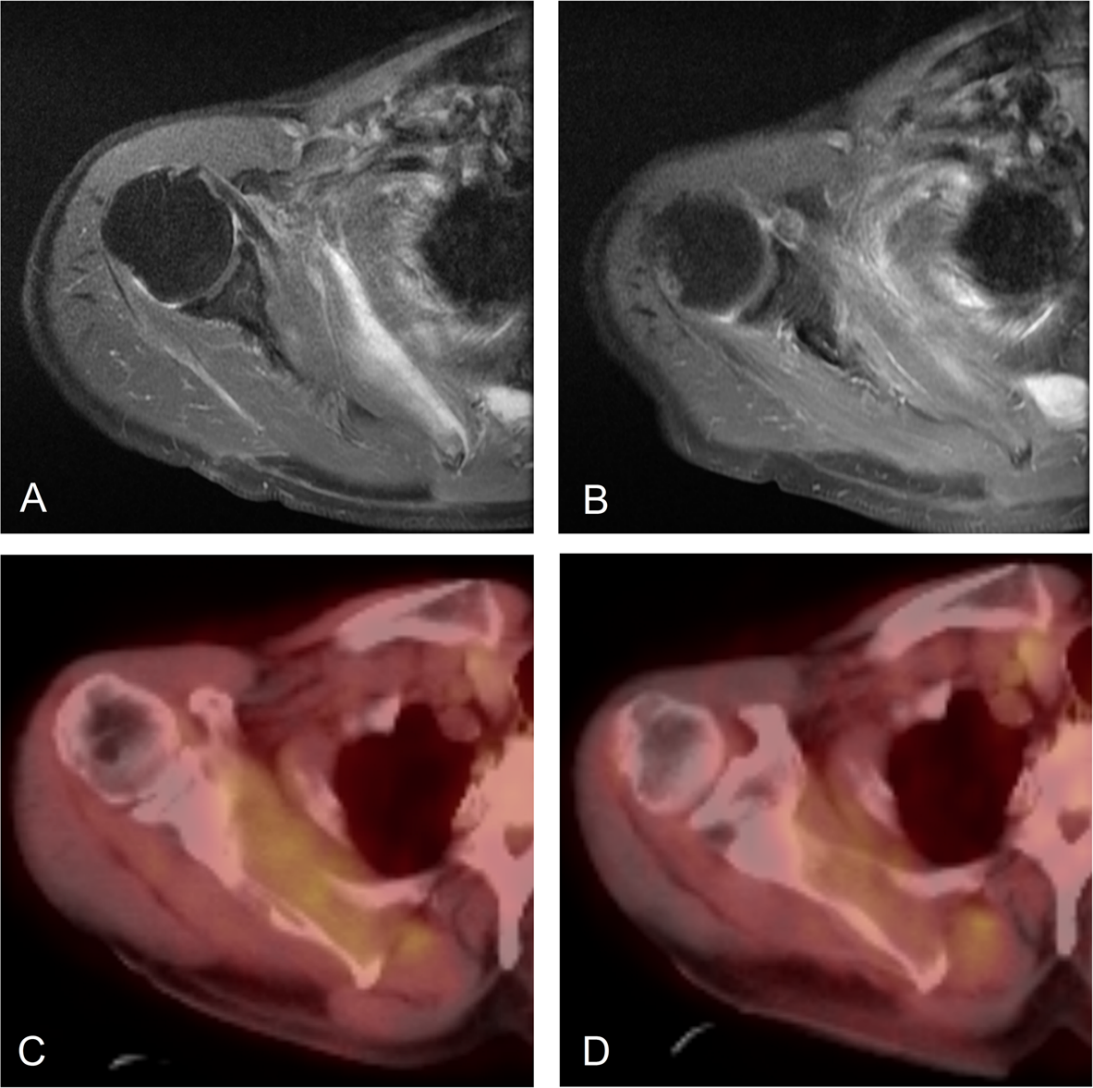
An undifferentiated sarcoma occurred in the chest wall of a 74-year-old male. A small enhanced lesion persisted three months after CIRT (**a**), and the size of the lesion increased on MRI performed five months after CIRT (**b**). FDG-PET/CT showed no change in the FDG accumulation between before (**c**) and after the recurrence (**d**)

**Fig. 2:**
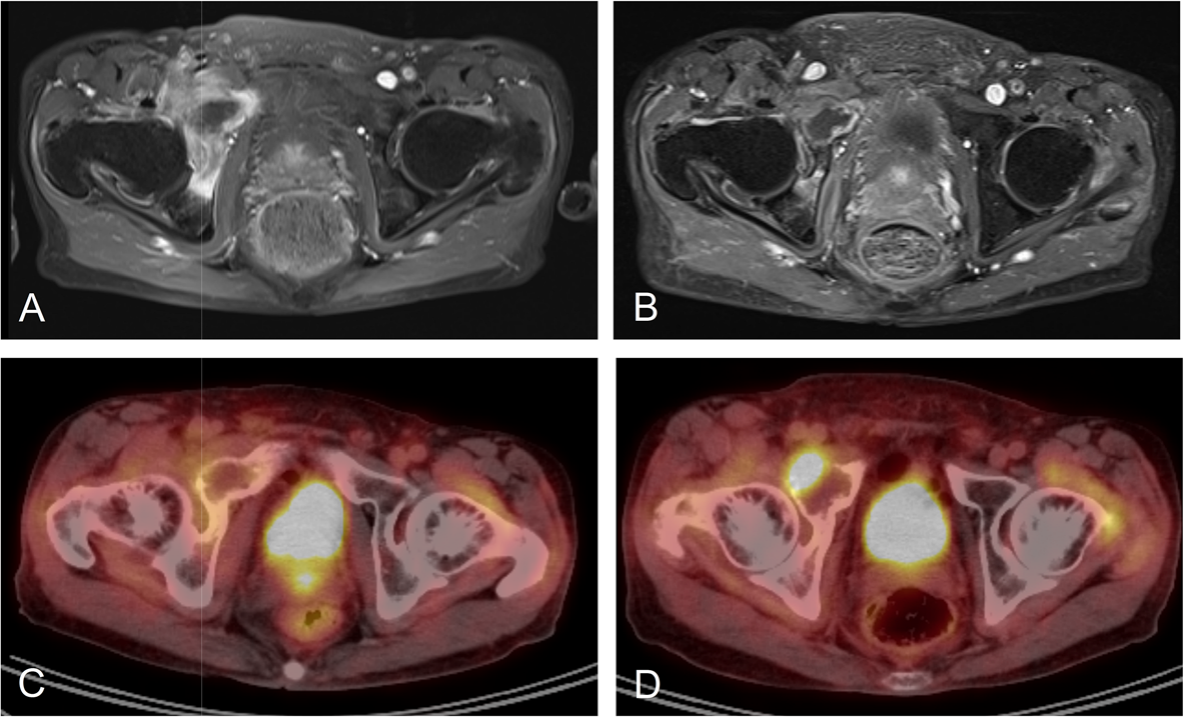
A recurrent tumor in a 79-year-old male with dedifferentiated chondrosarcoma. There were no mass lesions detected by enhanced MRI before (**a**) or after (**b**) the metastasis. CIRT induced a remission of the FDG accumulation noted by FDG-PET/CT three months after CIRT (**c**); however, a new lesion with high SUV appeared five months after CIRT (**d**)

Local recurrence appeared in 10 cases (27.0 %) after CIRT, with a mean duration of 13.9 (range: 5–25) months from treatment to relapse. Nine of the 10 lesions (90.0 %) were detected with FDG-PET/CT. Enhanced MRI was performed for 8 of the 10 cases, which detected 4 local recurrences (50.0 %). FDG-PET/CT found four local recurrences that enhanced MRI could not, while enhanced MRI could detect only one lesion that FDG-PET/CT missed (Fig. 1). Figure 2 shows the case with dedifferentiated chondrosarcoma, in which FDG-PET/CT could detect local recurrence after CIRT, although enhanced MRI could not. FDG-PET/CT and whole body CT detected distant metastasis in two cases.

### Evaluation of the predictors of local recurrence

There were no significant differences between the pre- and post-Tx tumor size (83.3 [16–180] mm vs 81.6 [15–172] mm, *p* = 0.29). All cases were classified to have stable disease. However, the SUVmax was significantly reduced three months after CIRT (Table 2). The differences in the values of the predictors for local recurrence between the recurrence and no recurrence groups are summarized in Table 3. Only the post-Tx SUVmax showed a significantly higher value in the recurrence group than in the no recurrence group. Among the examined predictors, there were no factors that were significantly superior for predicting local recurrence after CIRT (Table 4). Even the post-Tx SUVmax, which had the largest AUC of the various predictors examined, showed an insufficient sensitivity (70.0 %) and specificity (77.8 %), with an optimum cut-off value of 3.80.

**Table 2:** Difference in SUVmax and tumor size between pre- and post-CIRT

	pre-CIRT	post-CIRT	*p*-value
SUVmax	4.92 ± 3.68^a^	3.30 ± 2.08	**<0.01**
tumor size (mm)	83.27 ± 35.27	81.62 ± 35.97	0.29

*significant *p*-value was bold

^a^values were presented as mean ± standard deviation

**Table 3:** Difference in values of predictors between recurrence and no recurrence groups

	recurrence	no recurrence	*p*-value*
SUVmax in FDG-PET			
pre-Tx SUVmax	7.32 ± 5.47^a^	4.03 ± 2.32	0.10
post-Tx SUVmax	4.47 ± 2.94	2.86 ± 1.50	**0.03**
ΔSUVmax	2.85 ± 4.65	1.17 ± 1.73	0.29
ratio of SUVmax	0.83 ± 0.42	0.75 ± 0.34	0.56
tumor size			
pre-Tx tumor size	82.40 ± 39.04	83.60 ± 34.56	0.93
post-Tx tumor size	81.30 ± 36.68	81.74 ± 36.41	0.97
Δtumor size	1.10 ± 9.36	1.85 ± 9.60	0.83
ratio of tumor size	0.89 ± 0.35	0.97 ± 0.15	0.37

*Significant *p*-value was bold

^a^Values were presented as mean ± standard deviation

**Table 4:** Evaluation of the predictors for recurrence after CIRT with ROC curve

	AUC (95 % CI^a^)	*p*-value
SUVmax in FDG-PET		
pre-Tx SUVmax	0.65 (0.43–0.88)	0.16
post-Tx SUVmax	0.66 (0.44–0.88)	0.14
ΔSUVmax	0.54 (0.29–0.79)	0.71
ratio of SUVmax	0.57 (0.35–0.79)	0.57
tumor size		
pre-Tx tumor size	0.42 (0.20–0.64)	0.42
post-Tx tumor size	0.46 (0.25–0.67)	0.69
Δtumor size	0.54 (0.32–0.76)	0.73
ratio of tumor size	0.42 (0.20–0.64)	0.44

^a^ *CI* confidential interval

## Discussion

The current study showed that CIRT performed at our institute could treat inoperable bone and soft-tissue tumors with an acceptable recurrence rate as low as that in previous reports [8–10]. CIRT induced a significant decrease of FDG accumulation in the target tumors, and the post-Tx SUVmax in the recurrence group was significantly higher than that in the no recurrence group. These results suggested that CIRT could downregulate the tumor metabolism, and the tumors that exhibited decreased glucose transport activity following CIRT showed a reduced risk of local recurrence. Meanwhile, the tumor size was hardly changed after CIRT, and seemed to be inappropriate for evaluating the response to the treatment, which was consistent with previous reports showing that the SUV from FDG-PET/CT was superior to changes in tumor size for predicting tumor necrosis induced by neoadjuvant chemotherapy [12].

While a favorable effect of CIRT to reduce the tumor activity was shown, the present study also showed that accurately predicting the local recurrence of sarcomas after CIRT was difficult even when using the SUV to indicate the tumor metabolic activity. The presence of patients who had a relapse in spite of a decrease of the SUVmax in their lesions, and those who experienced no recurrence who did not show a change in their SUVmax after CIRT led to a low accuracy for predicting local recurrence with FDG-PET/CT. The probable reasons for the discrepancy between local recurrence and the tumor metabolism in some cases were as follow: First, most of the target tumors were composed of heterogeneous populations of cells. All of the recurrent tumors were detected as a much smaller lesion than the original tumors, which meant that a small population resistant to CIRT likely developed and appeared in PET and MRI as a recurrence, while most of the remaining original tumor was killed by CIRT. Second, there was a possibility that not only surviving tumor cells, but also remaining inflammatory cells in the treated lesion contributed to FDG accumulation. Previous reports speculated that inflammatory reactions evoked by radiation would increase the FDG uptake, and recommended that PET be performed more than six months and 12 weeks after radiotherapy to effectively detect residual colorectal tumors and non-small-cell lung cancers, respectively [16, 17]. In the current study, the earliest local recurrence occurred at five months after CIRT; and therefore, the prediction of the relapse would need to be performed earlier than that.

In clinical practice, early detection of the treatment response is important when providing follow-up for patients with malignant tumors, as the use of salvage and/or adjuvant treatment, even if relatively risky, should be considered as soon as possible if the risk of recurrence is high. However, the administration of additional therapy based on the findings of examinations with low specificity for detecting recurrence may worsen the patient’s status and quality of life without providing any major benefits.

Currently, there are no reliable modalities that can accurately predict the development of local recurrence after CIRT, and therefore, future studies should examine what method(s) is best to detect, not to predict, local recurrence in the early stage. In the current study, all but one case of local recurrence were detected using FDG-PET/CT; the remaining case was detected on enhanced MRI. The use of FDG-PET/CT is recommended to identify cases of local recurrence after CIRT for bone and soft-tissue tumors, and such evaluations are more accurate when combined with enhanced MRI.

There were two limitations associated with this study. First, the accumulation of long-term follow-up data was still insufficient, and therefore, we could not analyze whether PET/CT or any other modalities could predict the overall survival after CIRT. Second, we could not evaluate the risk factors or predictors of metastasis after CIRT because the number of metastases was too small to analyze. Recent reports have described the possibility that heavy ions affect molecules related to tumor metastasis and angiogenesis; and therefore, a further analysis on the relationship between metastasis and CIRT is required, although radiotherapies have been regarded as a local therapy for cancer [7]. These two problems can be resolved by the accumulation of more data and continuous follow-up.

## Conclusions

The combination of FDG-PET/CT and enhanced MRI is sensitive for detecting local recurrence and should be recommended for use in the follow-up of patients with sarcoma who receive CIRT, although there are currently no useful parameters of examinations performed before or three months after the initiation of treatment that can be used to accurately predict the development of local recurrence.

## Abbreviations

AUC: area under the curve
CIRT: carbon ion radiotherapy
GHMC: Gunma University Heavy Ion Medical Center
PET: positron emission tomography
RBE: relative biologic effectiveness
ROC: receiver operating characteristic
SUV: standardized uptake value
